# Association between atrial fibrillation, anticoagulation, risk of cerebrovascular events and multimorbidity in general practice: a registry-based study

**DOI:** 10.1186/s12872-016-0235-1

**Published:** 2016-03-28

**Authors:** Vigdis Vanbeselaere, Carla Truyers, Steven Elli, Frank Buntinx, Harrie De Witte, Jan Degryse, Séverine Henrard, Bert Vaes

**Affiliations:** Department of General Practice, KU Leuven (KUL), Kapucijnenvoer 33, blok j bus 7001, 3000 Leuven, Belgium; Department of General Practice, Maastricht University (UM), Maastricht, The Netherlands; Institute of Health and Society, Université Catholique de Louvain (UCL), Brussels, Belgium

**Keywords:** Atrial fibrillation, Multimorbidity, Cerebrovascular event, Anticoagulation, Aged

## Abstract

**Background:**

To date, there has been no comprehensive study on the association between atrial fibrillation (AF) and multimorbidity. The present study investigated the epidemiology of AF and the association between multimorbidity and the onset of AF. In addition, the correlation between multimorbidity and the use of anticoagulants and the risk of cerebrovascular events considering multimorbidity was explored in AF patients.

**Methods:**

Intego is a primary care registry network in Belgium. A case–control study was performed using Intego data from a 10-year time interval (2002 to 2011). All patients aged 60 years and older in 2002 who had developed new AF between 2002 and 2011 were selected, as well as a group of matched control patients. In addition, the prescription of anticoagulants and incident cerebrovascular events were recorded in patients with AF.

**Results:**

AF showed a prevalence of 5.3 % in 2002, and an upward trend was observed between 2002 and 2011. In all, 1830 patients with AF and 6622 control patients were included. AF patients had significantly more comorbidities (mCCI (modified Charlson Comorbidity Index) 5 ± 2 vs 4 ± 2, *P <* 0.001). In addition, 9.7 % of patients with AF developed a cerebrovascular event (mean follow-up time of 2.7 ± 2.5 years). Both the under- and overuse of anticoagulants was observed. Of the 49 % of patients with AF who were considered at high risk (CHADS_2_ ≥ 2), 50 % received anticoagulants in the first six months after diagnosis, whereas 49 % of patients who were at low risk (CHADS_2_ = 0) did not.

**Conclusions:**

AF is highly prevalent in older primary care patients and is significantly associated with multimorbidity. A discrepancy between the guidelines and clinical practice of anticoagulant use was observed. As multimorbidity seems to play a role in this, further qualitative research to study the perception and motives of the general practitioner is needed.

## Background

Atrial fibrillation (AF) is the most common sustained cardiac arrhythmia. The prevalence of AF is known to increase with age: more than three-quarters of patients with AF are older than 65 years [1]. AF currently affects more than 8.8 million adults in the European Union. This number is expected to double within the next 50 years as the population ages rapidly [2]. Patients with AF typically have multiple comorbidities and have a higher risk of cerebrovascular events compared with that of individuals without the condition [3]. As a result, knowledge of the interactions between comorbidity and AF, as well as good anticoagulation management are a primary challenge both for medical and economic reasons. General practitioners (GPs) play a key role in the patient-centred care of older patients with AF and concomitant conditions.

Oral anticoagulation treatment for high stroke-risk patients with AF has been demonstrated to be effective in reducing the risk of a stroke [4]. Stroke risk is assessed using the CHADS_2_ and CHA_2_DS_2_-VASc scores, validated stratification schemes in which the presence of certain comorbidities determines the need for anticoagulation treatment [5, 6]. Because increasing age itself is an important risk factor for a stroke, international guidelines recommend also using these scoring systems for older patients with AF [7, 8].

Nevertheless, many reports suggest genuine underuse of anticoagulants in elderly patients, even when correcting for the increased risk of bleeding in this age group [9, 10]. Increasing prevalence of multimorbidity in primary care complicates treatment [11]. To date, very few studies have assessed the link between AF, multimorbidity and stroke risk in older patients.

Therefore, this registry-based study investigated the epidemiology of AF and the association between multimorbidity and the onset of AF. In addition, the correlation between multimorbidity and the prescription of anticoagulants and the risk of cerebrovascular events considering multimorbidity over a 10-year time interval was explored in AF patients. The central hypothesis was that multimorbidity plays an important role in the aetiology of AF and in the treatment and future risk of cerebrovascular events for people who develop AF.

## Methods

### Study design

Data were obtained from Intego, a Flemish general practice-based morbidity registration network at the Department of General Practice of the University of Leuven [12]. Ninety-seven general practitioners (GPs), all using the medical software programme Medidoc (Corilus NV, Aalter, Belgium), collaborated in the Intego project. These 97 GPs work in 55 practices evenly spread over Flanders, Belgium. GPs applied for inclusion in the registry. Before acceptance of their data, registration performance was audited using a number of algorithms that compared their results with those of all other applicants. Only the data of the practices with an optimal registration performance were included in the database. The selection procedure has been described in detail previously [12]. The Intego GPs prospectively and routinely registered all new diagnoses together with new drug prescriptions, as well as laboratory test results and some background information (including gender and year of birth), using computer-generated keywords internally linked to codes. Using specially framed extraction software, new data were encrypted and collected from the GPs’ personal computers and entered into a central database. Registered data were continuously updated and historically accumulated for each patient. New diagnoses were classified according to a very detailed thesaurus automatically linked to the International Classification of Primary Care (ICPC-2) and International Statistical Classification of Diseases and Related Health Problems 10^th^ Revision (ICD-10). Drugs were classified according to the WHO’s Anatomical Therapeutic Chemical (ATC) classification system.

Intego started to collect data in 1994, but for the present study, data over a 10-year time interval from January 1^st^, 2002, to December 31^st^, 2011, were used.

### Study population

In 2002, 20,301 patients registered in the Intego database were aged 60 years and older and had at least had one contact with their general practitioner. Of these, 1068 had already been diagnosed with AF before 2002 and were therefore excluded from our study. The inclusion criterion for the cases was a reported first diagnosis of AF between January 1^st^, 2002 and December 31^st^, 2011. In total 1830 patients were diagnosed with AF. All participants were followed until the last contact date in the Intego registry or until December 31^st^, 2011, whichever came first.

Each patient with AF was matched with 3 to 4 control patients, bringing the number of controls up to 6622 [13]. These patients were still in the Intego database at the moment of the AF diagnosis in the case, belonged to the same age stratum in 2002 (i.e., 60–69, 70–79 or ≥80 years old), were of the same gender and originated from the same GP practice but had not been diagnosed with AF before or during the follow-up period.

### Clinical characteristics

#### Outcome variables

The first outcome variable was the diagnosis of AF. The date of diagnosis served as the baseline date for the case and its matched controls. The association between AF and comorbidities was explored in the entire group of cases and controls (*n* = 8452). A second outcome variable was the diagnosis of a cerebrovascular event (i.e., transient ischaemic attack (TIA) (ICPC – code K89) or cerebrovascular accident (ICPC – code K90)) in patients with AF after baseline. The risk of cerebrovascular events was calculated in the group of cases (*n* = 1830).

The internationally used definition of TIA changed in 2009, during our follow-up period. Whereas the temporary nature of neurologic symptoms (<24 h) were previously emphasized, a TIA is currently defined as a transient episode of neurological dysfunction caused by focal brain, spinal cord, or retinal ischaemia without acute infarction [14]. It is unclear which of both definitions the GPs used. Therefore, we here used ‘cerebrovascular event’ for both TIA and CVA.

CHADS_2_ and CHA_2_DS_2_-VASc (congestive heart failure, hypertension, age ≥75 years, diabetes mellitus, prior cerebrovascular event or thromboembolism, vascular disease (myocardial infarction or peripheral arterial disease), age 65–74 years and female gender) scores at baseline were calculated to determine the risk of a cerebrovascular event in patients with AF [5, 6].

#### Comorbidity

The Charlson Comorbidity Index (CCI) includes 19 chronic diseases, which are weighted based on their association with mortality [15]. The presence of these chronic diseases was not assessed for this study, but the history of chronic diseases is registered by the general practitioner in the electronic health record. A one-time registration before the baseline date was considered positive for cases and controls. The following diagnoses were included: diabetes mellitus; a history of myocardial infarction; heart failure; a history of cerebrovascular event; peripheral arterial illness; chronic pulmonary disease; a history of peptic ulcer; dementia; liver disease; hemiplegia; a history of cancer; a history of leukaemia; a history of lymphoma; and AIDS. Connective tissue disease could not be reliably assessed from the registry. Furthermore, the differentiation between cancers with or without metastasis, diabetes with or without end organ failure, and mild or moderate to severe liver disease could not be made. Consequently, all patients with any cancer history were assigned the same score (=2), as were all patients with diabetes (=1) and all patients with liver disease (=1). Therefore, we used a modified CCI (mCCI) in all further analyses [16].

The mCCI at the time of diagnosis of AF was calculated for each case and its controls. An mCCI was not available for 87 patients with AF because no creatinine levels were available in the database. In addition to the mCCI, diagnoses of hypertension and valvular heart disease were extracted at baseline.

#### Pharmacotherapy

The prescription of cardiovascular medication (ATC-coded) including beta-blockers, angiotensin-converting enzyme (ACE) inhibitors, angiotensin receptor blockers, calcium antagonists, diuretics, digitalis and antiarrhythmic drugs was extracted for every study participant at baseline and six months after the diagnosis of AF.

The prescription of antithrombotic therapy was registered for cases in the first six months after the diagnosis of AF (ATC-coded: heparin, acetylsalicylic acid, thienopyridine, dipyridamol, vitamin K antagonists). Throughout this paper we used the term ‘anticoagulants’ for both oral anticoagulants (vitamin K antagonists) and subcutaneous heparin treatment. When assessing whether patients were receiving anticoagulation treatment after the diagnosis of AF, we used a six-month time frame as there could be a doctor-related delay in registering treatment changes.

### Data analysis

Continuous data are presented as the mean and standard deviation (SD). Categorical data are presented as numbers and frequencies. Comparisons between cases and controls were performed using the independent samples *t*-test or the *χ*^2^ test for categorical data.

The prevalence and incidence of AF for each year were calculated in the yearly contact group. These included all the Intego patients of 60 years and older who had had at least one contact with their GP that year. Further analyses were made using the data from patients included in the case–control study.

The association between the presentation of novel AF and comorbidity was explored in the entire study population (*n* = 8452) by calculating odds ratios (ORs) with the corresponding 95 % confidence intervals (CIs) using bivariate and multivariable binary conditional logistic regression analyses adjusting for cardiovascular medication at the moment of diagnosis. Two different models were used; one with the mCCI and one with the different comorbidities of the mCCI without the overall index. These analyses were also performed in different age strata to see whether the pattern of multimorbidity in relation to AF changes between age groups.

The risk of cerebrovascular event after baseline in patients with AF (*n* = 1830) was calculated using a Cox proportional hazards model and adjusted for age and gender. Two different models were used: one with the mCCI and CHADS_2_ or CHA_2_DS_2_-VASc score and one with the different comorbidities without the overall index.

The association between the prescription of anticoagulants in the first six months after baseline and multimorbidity was investigated in patients with AF (*n* = 1830) using bivariate and multivariable binary logistic regression analyses adjusting for age, gender and cardiovascular medication prescribed in the six months after baseline. Two models were used: one with the mCCI and CHADS_2_ or CHA_2_DS_2_-VASc score and one with the different comorbidities without the overall index.

Subsequently, in patients with AF who developed a cerebrovascular event, individual patient profiles were investigated to explore further the relationship between the prescription of anticoagulation and the incidence of cerebrovascular events. For those who had received oral anticoagulation treatment (vitamin K antagonists), the last INR values before the cerebrovascular event were checked to see whether they were within a therapeutic range (INR 2–3.5) (cross section method for time-in-therapeutic range). Patients who had received a first diagnosis of AF at the same time as when they had had a cerebrovascular event were excluded from certain analyses because there had been no time to administer anticoagulants before the event.

To avoid co-linearity, the correlation coefficients between all covariates were calculated. In the case of co-linearity (Pearson’s r >0.80), only one of the two covariates was considered in the multivariable model. When clinically relevant, interaction was assessed between the variables used in the analyses. If the interaction term was statistically significant (*P* <0.05), separate models were run to assess the direction of association in different strata.

Statistical analyses were performed using SPSS 22.0 (SPSS Inc., Chicago, IL, USA).

## Results

### Epidemiology

**Fig. 1:**
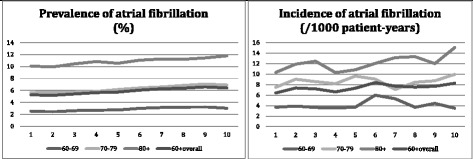
Prevalence and incidence of atrial fibrillation in the Intego database in patients of 60 years and older

Figure 1 shows the yearly prevalence of AF in the Intego database in patients aged 60 years or older from 2002 to 2011. An upward trend was observed with an overall prevalence of 5.3 % in 2002 compared with 6.4 % in 2011. The yearly incidence rate of AF is also shown in Fig. 1. The overall incidence rate rose slightly from 6.4/1000 patient-years in 2002 to 8.3/1000 patient-years in 2011. This upward trend was not present in the youngest patient group. AF incidence was highest in the oldest group of patients.

Our cohort included 8452 patients, of whom 1830 patients were diagnosed with AF between 2002 and 2011. The mean age was 77 years, and 49 % of our patients were men, as shown in Table 1.

**Table 1:** Baseline characteristics of patients with newly developed atrial fibrillation in the Intego database (2002–2011)^a^

	Atrial fibrillation	Control	*P* value^*^
	(*n* = 1830)	(*n* = 6622)	
Age (years), mean ± SD	77.5 ± 7.3	77.0 ± 7.2	0.006
Men, n (%)	900 (49.2)	3253 (49.1)	0.97
Cardiovascular medication before the diagnosis of atrial fibrillation, n (%)	1005 (54.9)	2523 (38.1)	<0.001
Diuretic, n (%)	495 (27.0)	1198 (18.1)	<0.001
β blocker, n (%)	603 (33.0)	1357 (20.5)	<0.001
Calcium channel blocker, n (%)	303 (16.6)	674 (10.2)	<0.001
ACE-inhibitor, n (%)	340 (18.6)	756 (11.4)	<0.001
ARB, n (%)	172 (9.4)	440 (6.6)	<0.001
Comorbidity			
Charlson Comorbidity Index, mean ± SD	5 ± 2	4 ± 2	<0.001
Myocardial infarction, n (%)	235 (12.8)	513 (7.7)	<0.001
Heart failure, n (%)	214 (11.7)	260 (3.9)	<0.001
Peripheral vascular disease, n (%)	149 (8.1)	400 (6.0)	0.001
Cerebrovascular event, n (%)	259 (14.2)	633 (9.6)	<0.001
Dementia, n (%)	62 (3.4)	242 (3.7)	0.59
Chronic pulmonary disease, n (%)	47 (2.6)	175 (2.6)	0.86
History of peptic ulcers, n (%)	150 (8.2)	472 (7.1)	0.12
Liver disease, n (%)	57 (3.1)	184 (2.8)	0.44
Diabetes, n (%)	319 (17.4)	884 (13.3)	<0.001
Hemiplegia, n (%)	30 (1.6)	71 (1.1)	0.048
Renal disease, n (%)	208 (11.9)	480 (8.1)	<0.001
History of tumours, n (%)	237 (13.0)	922 (13.9)	0.28
Leukaemia, n (%)	5 (0.3)	24 (0.4)	0.56
Lymphoma, n (%)	7 (0.4)	33 (0.5)	0.52
Hypertension, n (%)	780 (42.6)	2213 (33.4)	<0.001
Valve disease, n (%)	181 (9.9)	235 (3.5)	<0.001

^a^, the date of diagnosis of atrial fibrillation served as the baseline date for the case and its matched controls; ^*^, independent samples *t*-test or Chi^2^ test

*SD* standard deviation, *ACE* angiotensin-converting enzyme, *ARB* angiotensin receptor blocker

### Comorbidity

Cases differed significantly from controls for several parameters, such as the prescription of cardiovascular medication and the presence of comorbidity (Table 1). More specifically, patients with AF presented more often with a history of myocardial infarction, heart failure, peripheral vascular disease, a history of cerebrovascular events, diabetes, renal disease, hypertension and valvular heart disease. A total of 92 % of patients with AF had an mCCI of ≥3 versus 90 % of the controls (*P* = 0.004).

The mCCI was independently associated with a higher risk for AF (Table 2). In the model without the overall index, a history of myocardial infarction, heart failure, cerebrovascular event, renal disease and valvular heart disease were independently associated with AF. Hypertension was not independently associated, but antihypertensive treatment with β blockers, calcium channel blockers and ACE-inhibitors was associated with a higher AF risk.

**Table 2:** Association between comorbidity and the development of atrial fibrillation (binary conditional logistic regression analysis, *n* = 8452)

	Bivariate	*P* value	Multivariable	*P* value
	OR (95 % CI)		OR (95 % CI)	
Cardiovascular medication^a^				
Diuretic	1.7 (1.5–1.9)	<0.001	1.1 (0.94–1.3)	0.24
β blocker	2.0 (1.8–2.2)	<0.001	1.6 (1.4–1.8)	<0.001
Calcium channel blocker	1.8 (1.5–2.0)	<0.001	1.3 (1.1–1.5)	0.003
ACE-inhibitor	1.8 (1.6–2.1)	<0.001	1.3 (1.1–1.5)	0.008
ARB	1.5 (1.2–1.8)	<0.001	1.1 (0.85–1.3)	0.63
Comorbidity^a^				
Charlson Comorbidity Index (per point increase)	1.12 (1.08–1.16)	<0.001	1.09 (1.05–1.14)^b^	<0.001
Myocardial infarction	1.8 (1.5–2.1)	<0.001	1.3 (1.0–1.5)	0.021
Heart failure	3.3 (2.7–4.0)	<0.001	2.5 (2.0–3.1)	<0.001
Peripheral vascular disease	1.4 (1.1–1.7)	0.001	1.0 (0.82–1.3)	0.89
Cerebrovascular event	1.6 (1.3–1.8)	<0.001	1.3 (1.1–1.6)	0.003
Dementia	0.89 (0.66–1.2)	0.43	0.86 (0.62–1.2)	0.37
Chronic pulmonary disease	1.0 (0.71–1.4)	0.99	0.94 (0.66–1.3)	0.72
History of peptic ulcers	1.2 (0.96–1.4)	0.12	1.0 (0.82–1.3)	0.92
Liver disease	1.1 (0.83–1.5)	0.47	0.99 (0.72–1.4)	0.96
Diabetes	1.4 (1.2–1.6)	<0.001	1.1 (0.89–1.2)	0.58
Hemiplegia	1.5 (0.98–2.3)	0.058	1.1 (0.71–1.8)	0.61
Renal disease	1.5 (1.3–1.8)	<0.001	1.4 (1.1–1.7)	0.001
History of tumours	0.92 (0.79–1.1)	0.31	0.86 (0.73–1.02)	0.086
Leukaemia	0.77 (0.29–2.0)	0.59	0.65 (0.22–1.9)	0.44
Lymphoma	0.75 (0.33–1.7)	0.49	0.85 (0.36–2.0)	0.72
Hypertension	1.5 (1.4–1.7)	<0.001	1.1 (0.97–1.3)	0.15
Valve disease	3.1 (2.5–3.8)	<0.001	2.5 (2.0–3.1)	<0.001

^a^All variables were recorded at baseline (= date of the diagnosis of atrial fibrillation for the case and its matched controls); ^b^, Adjusted for diuretics intake, β blocker intake, calcium channel inhibitor intake, ACE inhibitor intake, ARB intake, arterial hypertension and valve disease. *OR* odds ratio, *CI* confidence interval, *ACE* angiotensin-converting enzyme, *ARB* angiotensin receptor blocker

The analyses were repeated with patients split into three age strata (Table 3). A history of heart failure and valvular heart disease were independently associated with AF in all age strata. Patients aged 60–79 years with AF were prescribed more β blockers than the controls. In patients aged 80 years and older, a strong and independent association was observed between AF and ACE-inhibitor prescription, diabetes, renal disease, a history of tumours and hypertension that was not observed in younger patients. However, the presence of diabetes and a history of tumours were negatively associated with AF diagnosis.

**Table 3:** Association of comorbidity and the development of atrial fibrillation within different age strata (binary conditional logistic regression analysis, *n* = 8452)

	60–69 years (2002)		70–79 years (2002)		≥80 years (2002)	
	Bivariate	Multivariable	Bivariate	Multivariable	Bivariate	Multivariable
Cardiovascular medication^a^						
Diuretic	1.6 (1.3–2.0)^*^	1.1 (0.82–1.4)	2.0 (1.7–2.4)^*^	1.3 (1.0–1.6)^$^	1.2 (0.86–1.6)	0.77 (0.54–1.1)
β blocker	2.2 (1.8–2.7)^*^	1.8 (1.5–2.3)^*^	2.0 (1.7–2.4)^*^	1.6 (1.3–1.9)^*^	1.4 (1.0–1.9)^$^	1.2 (0.80–1.7)
Calcium channel blocker	1.7 (1.3–2.2)^*^	1.2 (0.87–1.5)	2.1 (1.7–2.6)^*^	1.6 (1.3–2.0)^*^	1.1 (0.76–1.6)	0.89 (0.58–1.4)
ACE-inhibitor	1.7 (1.3–2.1)^*^	1.1 (0.86–1.5)	1.8 (1.5–2.3)^*^	1.2 (0.91–1.5)	2.1 (1.5–2.9)^*^	1.7 (1.1–2.6)^$^
ARB	1.8 (1.4–2.4)^*^	1.2 (0.89–1.7)	1.5 (1.1–1.9)^$^	0.89 (0.64–1.2)	0.82 (0.45–1.5)	0.81 (0.42–1.6)
Comorbidity^a^						
Charlson Comorbidity Index (per point increase)	1.2 (1.1–1.3)^*^	1.1 (1.0–1.2)^μ,$^	1.1 (1.1–1.2)^*^	1.1 (1.0–1.2)^μ,*^	1.0 (0.94–1.1)	1.0 (0.93–1.1)^μ^
Myocardial infarction	2.2 (1.6–2.9)^*^	1.4 (0.97–1.9)^£^	1.7 (1.3–2.1)^*^	1.2 (0.89–1.6)	1.6 (1.1–2.3)^$^	1.3 (0.89–2.0)
Heart failure	4.4 (2.8–7.0)^*^	3.0 (1.8–5.0)^*^	3.6 (2.7–4.8)^*^	2.8 (2.0–3.9)^*^	2.4 (1.8–3.4)^*^	2.2 (1.5–3.2)^*^
Peripheral vascular disease	1.6 (1.1–2.3)^$^	1.2 (0.79–1.8)	1.5 (1.1–2.0)^$^	0.93 (0.68–1.3)	0.99 (0.62–1.6)	0.83 (0.49–1.4)
Cerebrovascular event	2.1 (1.6–2.9)^*^	1.7 (1.2–2.4)^*^	1.7 (1.4–2.2)^*^	1.5 (1.2–1.9)^$^	0.98 (0.72–1.4)	0.84 (0.59–1.2)
Dementia	1.0 (0.33–3.0)	1.7 (0.51–5.3)	0.91 (0.60–1.4)	0.90 (0.56–1.4)	0.85 (0.55–1.3)	0.74 (0.45–1.2)
Chronic pulmonary disease	1.3 (0.75–2.4)	1.4 (0.77–2.6)	0.94 (0.57–1.6)	0.84 (0.50–1.4)	0.76 (0.37–1.6)	0.74 (0.34–1.6)
History of peptic ulcers	1.1 (0.75–1.5)	0.85 (0.58–1.2)	1.3 (0.97–1.7)^£^	1.1 (0.78–1.4)	1.1 (0.71–1.7)	1.1 (0.71–1.8)
Liver disease	1.7 (1.1–2.6)^$^	1.4 (0.87–2.2)	0.88 (0.55–1.4)	0.83 (0.50–1.3)	0.54 (0.20–1.5)	0.48 (0.17–1.4)
Diabetes	1.8 (1.4–2.3)^*^	1.3 (0.98–1.7)^£^	1.4 (1.2–1.7)^*^	1.1 (0.83–1.3)	0.72 (0.49–1.1)	0.60 (0.39–0.92)^$^
Hemiplegia	1.6 (0.66–3.8)	0.87 (0.31–2.5)	1.5 (0.78–2.7)	1.1 (0.53–2.1)	1.6 (0.69–3.5)	1.8 (0.77–4.4)
Renal disease	1.6 (1.0–2.5)^$^	1.1 (0.69–1.9)	1.6 (1.2–2.1)^*^	1.4 (1.0–1.8)^$^	1.4 (1.1–1.9)^$^	1.5 (1.1–2.1)^$^
History of tumours	0.92 (0.70–1.2)	0.83 (0.61–1.1)	1.1 (0.85–1.3)	1.0 (0.81–1.3)	0.63 (0.43–0.92)^$^	0.60 (0.40–0.91)^$^
Leukaemia	0.61 (0.07–5.1)	NA	0.85 (0.24–3.0)	0.87 (0.23–3.3)	0.72 (0.08–6.2)	0.97 (0.10–9.0)
Lymphoma	1.2 (0.25–6.1)	2.3 (0.36–15)	0.49 (0.14–1.7)	0.52 (0.15–1.8)	1.2 (0.24–5.9)	0.84 (0.14–4.9)
Hypertension	1.5 (1.2–1.8)^*^	1.0 (0.82–1.3)	1.6 (1.3–1.8)^*^	1.1 (0.91–1.3)	1.4 (1.1–1.8)^$^	1.4 (1.0–1.8)^$^
Valve disease	3.1 (2.0–4.7)^*^	2.4 (1.5–3.9)^*^	2.9 (2.2–3.9)^*^	2.2 (1.6–3.0)^*^	3.7 (2.4–5.8)^*^	3.3 (2.1–5.3)^*^

^a^All variables were recorded at baseline (= date of the diagnosis of atrial fibrillation for the case and its matched controls); ^*^, *P* < 0.001; ^$^, *P* < 0.05; ^£^, *P* < 0.10; ^μ^, adjusted for diuretics intake, β blocker intake, calcium channel inhibitor intake, ACE inhibitor intake, ARB intake, arterial hypertension and valve disease

*OR* odds ratio, *CI* confidence interval, *ACE* angiotensin-converting enzyme, *ARB* angiotensin receptor blocker

### Cerebrovascular event

A cerebrovascular event was diagnosed in 178 of the 1830 AF patients (9.7 %) during the 10-year time period (mean follow-up 2.7 ± 2.5 years). In total, 46 events (26 %) occurred on the same day as the incident AF, leaving 132 events occurring after the diagnosis of AF. In the first six months after baseline, 78 patients (44 %) developed a cerebrovascular event.

In both the bivariate and the multivariable analyses, age was associated with an incident cerebrovascular event in patients with AF (HR 1.05 (95 % CI 1.03–1.07), per year increase) (Table 4). Both CHADS_2_ and CHA_2_DS_2_-VASc scores were strongly associated with a cerebrovascular event (HR 1.5 (95 % CI 1.3–1.7) and HR 1.4 (95 % CI 1.2–1.6), per point increase, respectively). Patients with AF who had had a cerebrovascular event in the past were at a much greater risk of a recurrent ischaemic cerebrovascular event (HR 5.2 (95 % CI 3.8–7.2)). An independent correlation was also found between renal disease at baseline and future risk of cerebrovascular events (HR 1.3 (95 % CI 1.1–1.6)).

**Table 4:** Risk of a cerebrovascular event in patients with atrial fibrillation considering comorbidity (Cox regression analysis, *n* = 1830)

	Bivariate	*P* value	Multivariable	*P* value
	HR (95 % CI)		HR 95 % CI	
Age (years)^a^	1.06 (1.03–1.08)	<0.001	1.05 (1.03–1.07)	<0.001
Men	0.82 (0.61–1.1)	0.19	1.1 (0.76–1.5)	0.74
Comorbidity^a^				
CHADS_2_ score^b^	1.7 (1.5–1.9)	<0.001	1.5 (1.3–1.7)^c^	<0.001
CHA_2_DS_2_-VASc score^b^	1.5 (1.3–1.6)	<0.001	1.4 (1.2–1.6)^d^	<0.001
Charlson Comorbidity Index^b^	1.3 (1.2–1.4)	<0.001	1.1 (0.97–1.2)^c^	0.14
Myocardial infarction	0.87 (0.54–1.4)	0.55	0.64 (0.39–1.0)	0.075
Heart failure	1.2 (0.75–1.8)	0.48	0.90 (0.57–1.4)	0.65
Peripheral vascular disease	1.9 (1.2–2.9)	0.006	1.5 (0.94–2.3)	0.093
Cerebrovascular event	5.6 (4.1–7.5)	<0.001	5.2 (3.8–7.2)	<0.001
Dementia	1.9 (0.93–3.8)	0.081	1.0 (0.48–2.1)	0.99
Chronic pulmonary disease	0.24 (0.034–1.7)	0.16	0.17 (0.024–1.2)	0.081
History of peptic ulcers	0.83 (0.46–1.5)	0.53	0.74 (0.41–1.3)	0.32
Liver disease	1.6 (0.82–3.1)	0.17	1.4 (0.69–2.8)	0.36
Diabetes	1.2 (0.80–1.7)	0.44	1.3 (0.87–1.9)	0.21
Hemiplegia	1.6 (0.61–4.4)	0.32	0.63 (0.23–1.8)	0.38
Renal disease	1.4 (1.2–1.7)	<0.001	1.3 (1.1–1.6)	0.006
History of tumours	1.3 (0.90–2.0)	0.15	1.1 (0.74–1.7)	0.59
Leukaemia	3.3 (0.46–24)	0.23	3.1 (0.42–23)	0.27
Lymphoma	0.049 (0.0–2923)	0.59	0.0 (0.0–5.0 × e^261^)	0.96
Hypertension	0.98 (0.73–1.3)	0.92	0.94 (0.69–1.3)	0.69
Valve disease	0.89 (0.53–1.5)	0.65	0.81 (0.48–1.4)	0.44

^a^All variables were recorded at baseline (= date of the diagnosis of atrial fibrillation for the case); ^b^, per point increase; ^c^, adjusted for age, gender, valve disease and CHADS_2_ or Charlson Comorbidity index; ^d^, adjusted for age, valve disease and Charlson Comorbidity index (the correlation coefficient between the CHADS_2_ and the CHA_2_DS_2_-VASc score was 0.91)

*HR* hazard ratio, *CI* confidence interval, *ACE* angiotensin-converting enzyme, *ARB* angiotensin receptor blocker

### Anticoagulation treatment

Determinants for receiving anticoagulants within the first six months after baseline are shown in Table 5. Overall, 49 % (*n* = 895) of patients with AF in our study received anticoagulants. In total, 53 % of men with AF were prescribed anticoagulants, compared with 45 % of female patients with AF. When split into age groups, 49 % of patients aged 60–69 years (*n* = 140), 52 % of patients aged 70–79 years (*n* = 425) and 45 % of patients aged 80 years and older (*n* = 330) were prescribed anticoagulants.

**Table 5:** Determinants of receiving anticoagulants in the 6 months after the diagnosis of atrial fibrillation (binary logistic regression analysis, *n* = 1830)

	Bivariate	*P* value	Multivariable	*P* value
	OR (95 % CI)		OR 95 % CI	
Age (years)^a^	0.98 (0.96–0.99)	<0.001	1.00 (0.98–1.01)	0.74
Men	1.3 (1.1–1.6)	0.002	1.5 (1.2–1.8)	0.001
Medication^a^				
Digitalis	1.9 (1.4–2.5)	<0.001	2.0 (1.4–2.7)	<0.001
Antiarrhythmic drug	2.4 (1.9–3.0)	<0.001	2.0 (1.6–2.5)	<0.001
Antihypertensive medication				
Diuretic	2.5 (2.1–3.0)	<0.001	1.5 (1.2–1.9)	0.001
β blocker	3.1 (2.5–3.7)	<0.001	2.4 (1.9–2.9)	<0.001
Calcium channel blocker	2.2 (1.7–2.8)	<0.001	1.6 (1.2–2.1)	0.003
ACE-inhibitor	2.1 (1.7–2.6)	<0.001	1.5 (1.1–1.9)	0.003
ARB	3.0 (2.2–4.3)	<0.001	2.3 (1.6–3.4)	<0.001
Comorbidity^a^				
CHADS_2_ score^b^	1.0 (0.94–1.1)	0.64	1.1 (0.99–1.3)^c^	0.079
CHA_2_DS_2_-VASc score^b^	0.98 (0.92–1.0)	0.57	1.1 (0.99–1.3)^d^	0.086
Charlson Comorbidity Index^b^	0.91 (0.86–0.96)	<0.001	0.90 (0.83–0.97)^c^	0.009
Myocardial infarction	1.0 (0.79–1.4)	0.77	0.84 (0.61–1.2)	0.29
Heart failure	0.99 (0.74–1.3)	0.92	1.0 (0.72–1.4)	0.92
Peripheral vascular disease	1.2 (0.86–1.7)	0.30	1.2 (0.80–1.7)	0.41
Cerebrovascular event	1.1 (0.82–1.4)	0.66	1.2 (0.86–1.6)	0.32
Dementia	0.19 (0.097–0.38)	<0.001	0.26 (0.12–0.56)	<0.001
Chronic pulmonary disease	1.9 (1.0–3.4)	0.041	1.9 (0.97–3.7)	0.062
History of peptic ulcers	0.74 (0.53–1.0)	0.078	0.66 (0.45–0.96)	0.031
Liver disease	0.94 (0.55–1.6)	0.81	0.94 (0.51–1.7)	0.85
Diabetes	1.2 (0.96–1.6)	0.11	1.1 (0.85–1.5)	0.40
Hemiplegia	0.60 (0.28–1.3)	0.18	0.77 (0.33–1.8)	0.56
Renal disease	0.84 (0.73–0.97)	0.021	0.88 (0.74–1.0)	0.14
History of tumours	0.70 (0.53–0.93)	0.013	0.67 (0.49–0.92)	0.012
Leukaemia	0.26 (0.029–2.3)	0.23	0.36 (0.038–3.5)	0.38
Lymphoma	1.4 (0.31–6.2)	0.66	0.93 (0.19–4.5)	0.92
Hypertension	1.3 (1.0–1.5)	0.016	1.0 (0.82–1.3)	0.86
Valve disease	1.2 (0.88–1.6)	0.24	1.2 (0.87–1.8)	0.24
Cerebrovascular event in the first 6 months after diagnosis of atrial fibrillation	1.6 (1.0–2.6)	0.042	1.8 (1.0–3.1)	0.039

^a^ All variables were recorded at baseline (= date of the diagnosis of atrial fibrillation for the case), except cerebrovascular event in the first 6 months after the diagnosis of atrial fibrillation; ^b^, per point increase; ^c^, adjusted for age, gender, digitalis intake, antiarrhythmic drug intake, diuretic intake, β blocker intake, calcium channel blocker intake, ACE inhibitor intake, ARB intake, valve disease, cerebrovascular event in the first 6 months after diagnosis of atrial fibrillation and CHADS_2_ or Charlson Comorbidity index; ^d^, adjusted for age, digitalis intake, antiarrhythmic drug intake, diuretic intake, β blocker intake, calcium channel blocker intake, ACE inhibitor intake, ARB intake, valve disease, cerebrovascular event in the first 6 months after diagnosis of atrial fibrillation and Charlson Comorbidity index (the correlation coefficient between the CHADS_2_ and the CHA_2_DS_2_-VASc score was 0.91)

*OR* odds ratio, *CI* confidence interval, *ACE* angiotensin-converting enzyme, *ARB* angiotensin receptor blocker

Table 6 describes the prescription of anticoagulants according to the CHADS_2_ and CHA_2_DS_2_-VASc score. On the one hand, an overuse of anticoagulants was seen in patients with a low risk of cerebrovascular events (51 % of patients with a CHADS_2_ score of 0 were prescribed anticoagulants), and on the other hand an underuse of anticoagulants was seen in patients with a high risk of cerebrovascular events (50 % of patients with a CHADS_2_ score ≥2 were not prescribed anticoagulants). Overall, there was no significant association between the CHADS_2_ (*P* = 0.079) or CHA_2_DS_2_-VASc score (*P* = 0.086) and receiving anticoagulants after baseline.

**Table 6:** Prescription of anticoagulants according to the CHADS_2_ and CHA_2_DS_2_-VASc score in the first six months after the diagnosis of atrial fibrillation (*n* = 1830)

	CHADS_2_ score		
	0 (*n* = 285, 15.5 %)	1 (*n* = 654, 36 %)	≥2 (*n* = 891, 48.5 %)
Anticoagulants, n (%)	146 (51)	306 (47)	443 (50)
No anticoagulants, n (%)	139 (49)	348 (53)	448 (50)
	CHA_2_DS_2_-VASc score		
	0 (*n* = 19, 1 %)	1 (*n* = 157, 9 %)	≥2 (*n* = 1654, 90 %)
Anticoagulants, n (%)	11 (58)	81 (52)	803 (49)
No anticoagulants, n (%)	8 (42)	76 (48)	851 (51)

Of the 132 patients who developed a cerebrovascular event during the follow-up period, 34 (26 %) were taking anticoagulation therapy at the time of their event. Of these, 15 were within a therapeutic INR range (2–3.5) at the time of the last blood test before the event, 13 were controlled suboptimally (INR <2), whereas 3 had received more than optimal anticoagulation treatment (INR >3.5). For 3 patients receiving anticoagulants after the diagnosis of AF, no INR values were available. At the time of their cerebrovascular event, 98 patients were not receiving anticoagulants. Of these, 14 (14 %) were prescribed anticoagulants after the event.

## Discussion

This study showed AF was highly prevalent in older primary care patients and was significantly associated with multimorbidity. Multimorbidity was positively associated with both AF development and cerebrovascular event risk in patients with AF. A discrepancy between guidelines and clinical practice in anticoagulation treatment was observed. There was both under- and overuse of anticoagulants in patients with AF, possibly due to the multimorbidity of these patients, complicating treatment.

### Epidemiology

The prevalence of AF in our study population is comparable to the results of the Dutch Rotterdam Study, a population-based prospective cohort study between 1990 and 2000 in patients aged 55 years and older [1]. They calculated an overall AF prevalence of 5.5 %, whereas our overall prevalence was 5.3 % in 2002. The overall incidence in the Rotterdam population was 9.9/1000 patient-years, which was higher than the incidence in our population. Both studies found a steep increase in both prevalence and incidence with age.

An upward trend was observed in AF prevalence between 2001 and 2011. A study on the Global Burden of Disease 2010 data confirms this modest upward trend within the same time frame [17]. Several papers predict a more dramatic escalation in AF burden in the coming decades, both from a health care and whole-societal perspective [2, 18, 19].

### Comorbidity

The results of our study exceed previous findings of the high comorbidity index in patients with AF. The National Health and Wellness Survey data [3] from 2009 showed 80 % of American patients with AF had a CCI of 0–2, and 21 % had scores ≥ 3 compared with 8 and 92 %, respectively in our population. However, with a mean age of 65 years, their population was younger than our study population, which might explain the higher burden of comorbidities in our study. Moreover, their study was based on self-reports and thus possibly under-reported data from an internet-based survey, which might have excluded less computer-literate individuals. However, findings of both studies were comparable with regards to stroke-risk estimation with CHADS_2_ scores.

The current study found hypertension treatment, heart failure, myocardial infarction, valvular heart disease and renal disease to be independently associated with an increased risk of new-onset AF, in line with very recent statistical updates of AHA [20]. The relation between AF and comorbidity existed even in the oldest patients. The health burden carried by patients often extends far beyond AF and specifically does so in older persons.

### Cerebrovascular event

Almost 10 % of the patients with AF developed a cerebrovascular event during the 10-year time interval, emphasizing the importance of the thrombo-embolic risk. Although a history of cerebrovascular events before the diagnosis of AF did not significantly alter anticoagulation frequency, it was identified as a major risk factor for a recurrent event. This is similar to the results of a systematic review published by, ‘The Stroke Risk in Atrial Fibrillation Working Group’ [21]. A prior stroke proved to be the most powerful risk factor and reliably confers a high stroke risk in their study. Both CHADS_2_ and CHA_2_DS_2_-VASc scores were strongly associated with the risk of cerebrovascular events in our patients with AF. The use of these scores is mandatory in daily primary care practice. It might also be important to be extra careful with patients with AF and concomitant kidney problems, as our study observed a high cerebrovascular event risk in persons with renal disease.

Of the patients with AF who developed a cerebrovascular event in our study, 26 % did so at the time of first diagnosis of AF. Although it is possible that they developed an event immediately after the start of their heart rhythm disorder, there might also be a doctor-related delay in diagnosing heart rhythm problems in primary care patients.

### Anticoagulation treatment

Several studies have reported the underuse of anticoagulants in older patients and increasing age has been reported as a barrier to starting anticoagulants [1, 9, 10]. However, the current study showed that age, when corrected for comorbidity, was not a significant determinant of (not) receiving anticoagulants. This finding is in line with the guidelines, which state that even for the very elderly, the same risk-benefit analysis should be made to determine the need for anticoagulants [7, 8, 22, 23].

A literature review has identified both patient- and physician-related barriers to anticoagulation prescription [24]. We observed both overtreatment in low stroke-risk patients with AF and undertreatment in patients at high stroke risk when compared with the guidelines. CHADS_2_ and CHA_2_DS_2_-VASc scores were not significantly associated with anticoagulation prescription in our study. Of the patients at low risk for a stroke more than 50 % received anticoagulants. The same trend can be observed in other studies, such as the ORBIT-AF study [25]. Although it is possible that these patients may have received anticoagulants for conditions other than AF, it is unlikely that these conditions alone would account for the relatively high anticoagulation rates. GPs may be prescribing anticoagulants for these patients simply because they have AF, regardless of their low stroke risk. On the contrary, only 50 % of the individuals with a high risk of stroke (CHADS_2_ ≥ 2) were receiving anticoagulants. This is comparable to findings in other studies [3, 26]. It is possible that GPs are not aware of how infrequently they prescribe anticoagulants. Therefore, it is important that quality of care audits can easily be performed with their medical software programme.

Another reason for under-anticoagulation could be the bleeding risk of a patient with AF, as perceived by the GP. Patients with a history of peptic ulcers or a history of tumours received less anticoagulants, probably due to a high perceived bleeding risk. Patients suffering from dementia also received less anticoagulants. Fear of poor compliance and risk of falls might play a role in this.

The perceived thrombo-embolic risk of patients with AF could be a physician-related barrier to anticoagulation prescription. For example, male patients with AF in our Intego population more frequently received anticoagulants than women did. This finding is consistent with results from other studies [10]. Gender inequalities have been observed in the use of therapies in other areas of cardiovascular medicine and have been attributed to a possible lower perceived risk of cardiovascular disease in women compared with men, leading to an underrecording of risk factors and lower rates of prophylactic treatment in women [27]. However, the female gender is a component of the CHA_2_DS_2_-VASc score because epidemiologic data have shown that women have an increased thrombo-embolic risk compared to men [6].

Much research on anticoagulation in patients with AF has already been done and clinically useful guidelines have been developed [4, 7–10, 22, 24–26]. Thorough knowledge of the guidelines by the GPs is important to make evidence-based decisions. However, our study illustrates that there is still a discrepancy between the guidelines and clinical practice for stroke prevention in older patients with AF. It is important to clarify further the barriers that GPs experience when treating older patients with AF. As multimorbidity seems to play a role in the therapeutic decision-making process and in the risk of a stroke, it is important to understand fully its impact. Therefore, more qualitative research, such as focus group research or semi-structured interviews, needs to be performed to further investigate attitudes of GPs in prescribing anticoagulants for patients with AF and multimorbidity. Care plans should be designed and implemented according to the individual medical history, risk factors and needs of each patient. Primary care physicians are probably in the best position to take the key role in managing the care of our older patients.

### Strengths and limitations

A strong point of this study is the inclusion of a large primary care population, representative of the population in Flanders. In Belgium, the general practitioner is the central actor in the health care system. More than 95 % of people aged 60 and over have a regular general practitioner, and >90 % have at least one contact with their general practitioner every year [28]. The database contains all introduced diagnoses and most of the relevant clinical parameters. Due to the retrospective design, there was a 10-year follow-up of the clinical and biological parameters. Data analyses with longitudinal models incorporated between-subject (i.e., case – control) and within-subject analyses with the inclusion of timely changes in diagnoses and drug prescriptions. Therefore, we were able to perform the first comprehensive study about AF and multimorbidity in a primary care setting.

The limitations were the lack of mortality data and the lack of a creatinine value for <5 % of the included patients with AF. Due to database limitations, data to calculate HASBLED-scores, estimating the bleeding risk of a patient with AF, were not available. Recently, anticoagulation-therapy options have changed due to the introduction of the NOACs (i.e., factor Xa and thrombin inhibitors). These were not taken into account because the drugs were not yet available in Belgium before 2012.

## Conclusion

With a prevalence of 6.4 % in patients aged 60 years and older in 2011, AF is a very frequent condition in elderly primary care patients and a major risk factor for cerebrovascular events. Due to ageing of the population, AF prevalence is expected to rise in the coming decades, making preventative strategies to reduce the risk of AF within comprehensive health management programmes increasingly important. Our study confirms the very high burden of comorbidities in patients with AF. These conditions are predictors of both AF development and cerebrovascular event risk in patients with AF and should thus be considered when making decisions about anticoagulation treatment. There is both under- and overuse of anticoagulants in patients with AF, possibly due to the multimorbidity of these patients, complicating treatment. Further qualitative research is needed to clarify the nature of this correlation and of the barriers GPs experience when considering anticoagulation treatment. A better knowledge of these interactions could lead to improved preventive and curative management of the health of older patients.

### Ethics approval

The Intego procedures were approved by the ethical review board of the Medical School of the Catholic University of Leuven (no ML 1723) and by the Belgian Privacy Commission (no SCSZG/13/079).

### Provenance and peer review

Not commissioned; externally peer reviewed.

### Data sharing statement

All authors had full access to all of the data (including statistical reports and tables) in the study and can take responsibility for the integrity of the data and the accuracy of the data analyses.
